# Indigo Naturalis regulates the gut microbiota to increase SCFAs content and improve ulcerative colitis lesions

**DOI:** 10.3389/fcimb.2026.1772977

**Published:** 2026-03-31

**Authors:** Xiang-Yi Zhan, Ran Xu, Li-Zhi Hu, Zi-Xu Zhang, Ming-Sheng Zhou, Hui Jia

**Affiliations:** 1 School of Traditional Chinese Medicine, Shenyang Medical College, Shenyang, China; 2 Department of Pathology and Pathophysiology, School of Basic Medical Sciences, Shenyang Medical College, Shenyang, China; 3 Science and Experimental Research Center of Shenyang Medical College, Shenyang, China; 4 Shenyang Key Laboratory of Vascular Biology, Shenyang, China

**Keywords:** GM, indigo, Indigo naturalis, SCFAs, uc

## Abstract

**Background:**

The global prevalence of ulcerative colitis (UC) is approximately 5 million, and the incidence is on the rise. The gut microbiota (GM) plays a key role in the pathogenesis and progression of UC. Studies have found that Indigo Naturalis (abbreviated as Indan) and Indigo can regulate GM to improve UC, but the mechanism has not been elucidated.

**Materials and methods:**

Using 16S rDNA to examine GM changes and evaluate the effects of Indan/Indigo treatment on short-chain fatty acids (SCFAs).

**Results:**

The community structure of GM, there is a significant difference between the UC group and the control group. Among them, the abundance of the Proteobacteria phylum increases in the UC group, while the abundance of the Firmicutes phylum decreases. The dynamic balance between *Lachnospiraceae_unclassified* and *Escherichia* is a key factor in the pathogenesis of UC. Indan and indigo (Principal components of Indan) can alter the abundance and structure of GM. Indan and indigo increase the abundance of beneficial bacteria, leading to an increase in SCFAs content. Compared with indigo, Indan has a stronger effect on increasing the content of SCFAs. Indan can significantly increase the abundance of *Clostridium_sensu_stricto_1*, produce hexanoic acid. It is worth noting that the proportion regulation of Indan and indigo on the microbial structure of different phenotypes is also a new mechanism for the treatment of UC.

**Conclusion:**

Indan/Indigo regulates GM abundance and structure, enhances SCFAs content, inhibits inflammatory response, promotes ulcer healing, restores intestinal wall integrity, and treats UC.

## 1 Introduction

Ulcerative colitis (UC) is a chronic inflammatory bowel disease, characterized by diarrhea, mucous pus stools, abdominal pain, and weight loss. In recent years, patients with UC have a higher risk of developing colorectal cancer (CRC) than other populations, and it is considered a precancerous lesion of colorectal cancer (Segal et al., 2021). Currently, the first-line treatment options include amino-salicylate preparations, immunosuppressants, and biological agents (Singh et al., 2024). However, about 15% of patients still face the risk of UC recurrence (Wangchuk et al., 2024). Therefore, there is still an urgent need for effective and well-tolerated treatment strategies.

UC progression is closely related to the intestinal microenvironment and the integrity of the intestinal barrier (Dahiya et al., 2022). Intestinal barrier function is impaired in UC patients, leading to increased intestinal permeability and easier invasion of harmful substances and pathogens, exacerbating inflammatory reactions (Krugliak Cleveland et al., 2022). Damage to the intestinal barrier also leads to dysbiosis of gut microbiota (GM). GM dysbiosis exacerbates inflammation and UC progression by producing harmful substances, over-activating the immune system, and destroying the intestinal mucosal barrier. Short-chain fatty acids (SCFAs) are important metabolic products of GM, which are small molecular organic acids produced during the fermentation of dietary fibers by microorganisms, mainly including acetic acid, propionic acid, butyric acid (Cuervo-Zanatta et al., 2023). SCFAs can reduce intestinal pH and oxygen content, inhibit the growth of pathogenic bacteria, and promote the growth of probiotics (Luqman et al., 2024). At the same time, SCFAs are also an important source of energy for intestinal cells, which can maintain the functions of intestinal cell growth, repair, barrier, and immune regulation, and are of great significance for maintaining the stable intestinal environment.

Indigo Naturalis (Abbreviated as Indan) is a processed product derived from the leaves or leafy stems of Chinese herbs such as *Strobilanthes cusia* (Nees) Kuntze, *Persicaria tinctoria* (Aiton) Spach, and *Isatis tinctoria L*. Indan and its compound preparations show a stable efficacy in the treatment of UC and can effectively prevent the recurrence of UC (Kakdiya et al., 2024). Indigo is one of the main components of Indan, accounting for 5% of Indan and is relatively accessible. At the same time, Indigo, as a ligand of the aromatic hydrocarbon receptor, plays a certain role in immune and gut microbiota regulation (Xie et al., 2023). This study clarifies the mechanism of Indan/Indigo treatment for ulcerative colitis by studying the pathways by which Indan/Indigo regulates GM and SCFAs.

## 2 Material and methods

### 2.1 Laboratory animals and experimental grouping

Select healthy C57BL/6 mice (male, 6 weeks old, 18~22 g, Vital River Laboratory Animal Technology Co., Ltd., Beijing). Prior to the commencement of the experiment, the mice underwent a one-week acclimatization period. Following this period, the mice were randomly divided into four groups (n = 10): control group, model group, indan group, and indigo group. Random number table method was used to group mice to ensure that there were no significant differences in weight, age, and other characteristics between the groups. (Ethics Committee of Shenyang Medical College, Approval no. SYYXY2023120801).

### 2.2 Model construction

The UC model was developed utilizing 3% dextran sulfate sodium (DSS) aqueous solution (Mo et al., 2022). Mice in the model group, indan group, and indigo group were provided free access to the DSS solution for 7 days. During this period, variations in body weight, fecal characteristics, and water consumption of the mice were recorded. Following the DSS treatment, the efficacy of the UC model was assessed by measuring parameters such as the rate of weight loss and the characteristics of feces in the mice (Li et al., 2022).

### 2.3 Pharmacological intervention

**Figure 1:**
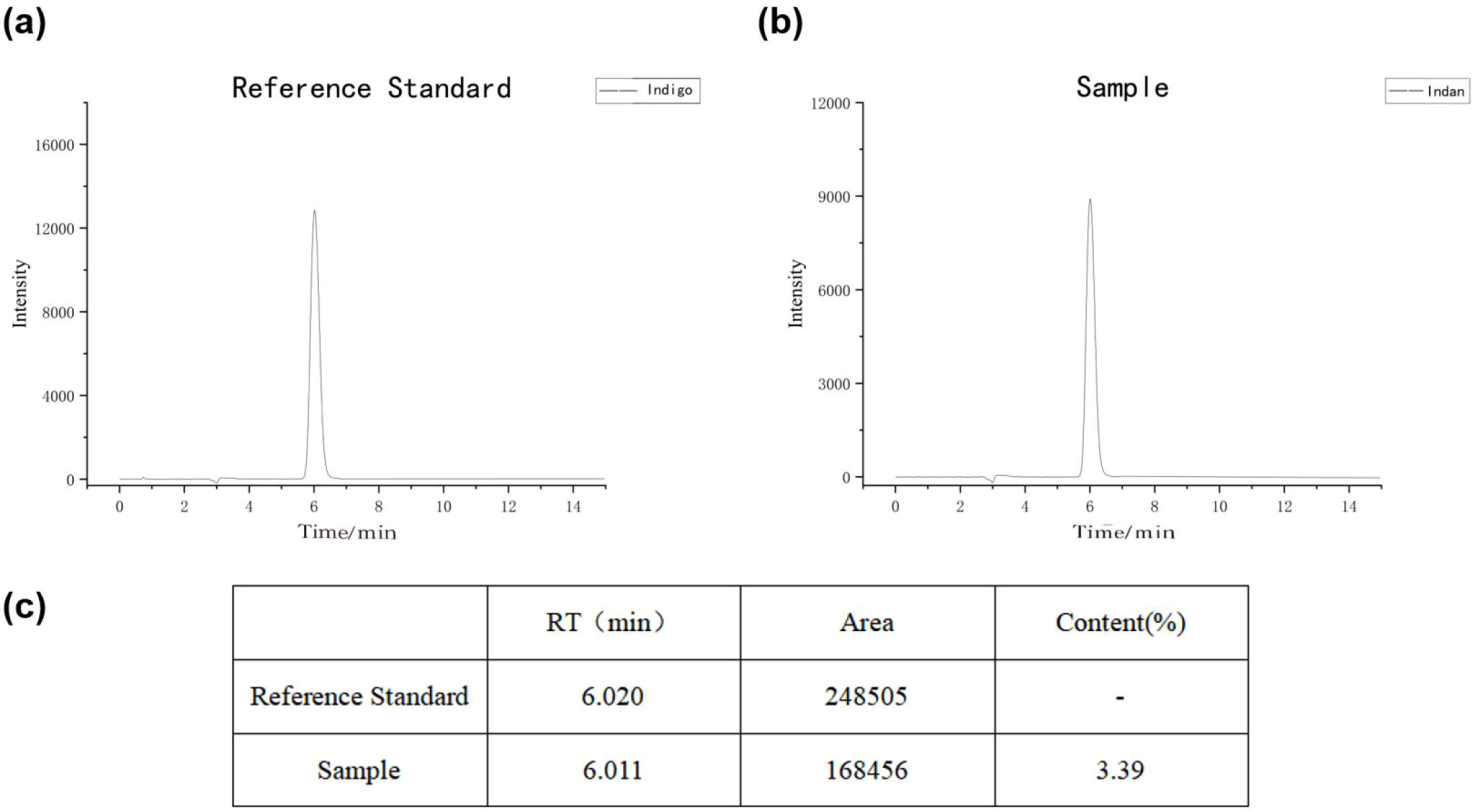
Indigo content detection. **(A)** Reference standard; **(B)** Sample; **(C)**Indigo content of the sample. RT, retention time; Area, peak area; Content, Indigo content of the sample.

The content of Indigo in Indan detected by HPLC was 3.39%(**Figures 1A–C**). Detection conditions: chromatographic column: TSK-GEL^®^, injection volume: 10 μL, flow rate: 1.0 mL/min, column temperature: 35°C, gradient elution: C: methanol D: water. Sample processing: take about 50 mg of Indan powder, add about 220 mL of 2% chloral hydrate chloroform solution, sonicate for 30 minutes, add 2% chloral hydrate chloroform solution to 250 mL, shake well, filter, and take the filtrate to obtain. Standard preparation: take 2.5 mg of indigo reference standard, add about 220 mL of 2% chloral hydrate chloroform solution, sonicate for 1.5 hours, add 2% chloral hydrate chloroform solution to 250 mL, shake well, and obtain (10 μg indigo per 1 mL).

Following the successful construction of the UC model, mice assigned to the Indan group and the Indigo group received Indan extract (400 mg/kg via gavage; the commonly used clinical dosage for adults in India is 2.5 g, and the dosage for mice was calculated using the body surface area conversion method) and indigo extract (15 mg/kg via gavage; the content of indigo in Indan is approximately 3.4%), respectively. Mice in the control and model groups were administered equivalent volumes of physiological saline *via* gavage. Throughout the treatment duration, data on weight fluctuations, fecal characteristics, and water consumption of the mice were recorded. The treatment period extended over 2 weeks. After the treatment, all animals were euthanized (Place the mice in an anesthesia canister and continue inhaling 3% isoflurane inhibition for 2–3 minutes. Once the mice have lost consciousness, euthanize them by cervical dislocation), colon tissue and colon contents were collected for subsequent detection and analysis.

### 2.4 Assessment of the UC disease activity index

The Disease Activity Index (DAI)= (weight loss score + stool consistency score + fecal bleeding score)/3 (Ruan et al., 2024), the table presents the scores corresponding to each item (**Table 1**). Fecal occult blood (FOB) testing conducted using the benzidine assay technique.

**Table 1:** DAI score allocation.

Scoring Item	Scoring Criteria
Weight Loss Score	0: No weight change
	1: 1–5% weight loss
	2: 5–10% weight loss
	3: 10–20% weight loss
	4: Weight loss exceeding 20%
Stool Consistency Score	0: Normal
	1: Soft stool
	2: Mucous stool
	3: Watery stool
Fecal Bleeding Score	0: FOB (-)-No bleeding
	1: FOB (+)-Blue color appears within 30–60 seconds
	2: FOB (++)-Immediately display blue-green color
	3: FOB (+++)-Immediately display deep blue
	4: Gross bloody

### 2.5 Histopathological examination

Colon tissue specimens were preserved in 10% neutral formalin solution for 24 h. The fixed colon tissue was dehydrated using gradient ethanol solution. Make the colon tissue transparent with xylene. Place the colon tissue in melted paraffin for wax immersion treatment. Subsequently, the colon tissue was embedded in paraffin, and sections were cut to a thickness of 5 μm, ensuring that each section encompassed the entirety of the colon tissue. Sections were stained with hematoxylin eosin (HE), and pathological alterations were assessed by microscopy.

### 2.6 Quantitative assessment of SCFAs

Collect mice feces after treatment. Weigh 50 mg of mice intestinal contents and add to 80% methanol-water for grinding. Take 10 μL of supernatant, add EDC solution and 3-NPH for derivatization. Add the initial mobile phase solution to 500 μL, vortex mix, and use for LC-MS/MS analysis. Chromatography-mass spectrometry conditions: column: ACQUITY UPLC BEH C_18_ (1.7 μm, 2.1 × 100 mm); injection volume: 2 μL; mobile phase: water: methanol + acetonitrile (1:1); mass spectrometry conditions: multiple reaction monitoring, negative ion mode. Determine the content of acetic acid, propionic acid, butyric acid, valeric acid, isobutyric acid, isovaleric acid, and hexanoic acid in the feces (Kukaev et al., 2024). Use principal component analysis (PCA) to study the overall differences in the SCFAs metabolic profiles of different groups of mice.

### 2.7 16S rDNA sequencing analysis

After the mice were euthanized, the contents of their colons were collected as test samples. Extract DNA from samples, perform PCR amplification, purify the product, and conduct on-machine sequencing to obtain raw data. Use overlaps to concatenate the paired-end data and perform quality control and chimera filtering to obtain high-quality clean data (Gall-David et al., 2023). Use Amplicon sequence variants (ASVs) to construct an Operational Taxonomic Units (OTUs) table, obtain the final ASV feature table and feature sequences, and further perform diversity analysis, species classification annotation, and differential analysis (García-López et al., 2021).

### 2.8 Statistical analysis

The experimental data were analyzed utilizing SPSS version 27.0, with quantitative data presented as mean ± standard deviation (mean ± SD). The comparison of means across multiple groups was executed through one-way analysis of variance (ANOVA), followed by *post hoc* testing employing Tukey’s HSD method. *p* < 0.05 was statistically significant.

## 3 Results

### 3.1 Indan improves the clinical symptoms and pathological changes of UC

**Figure 2:**
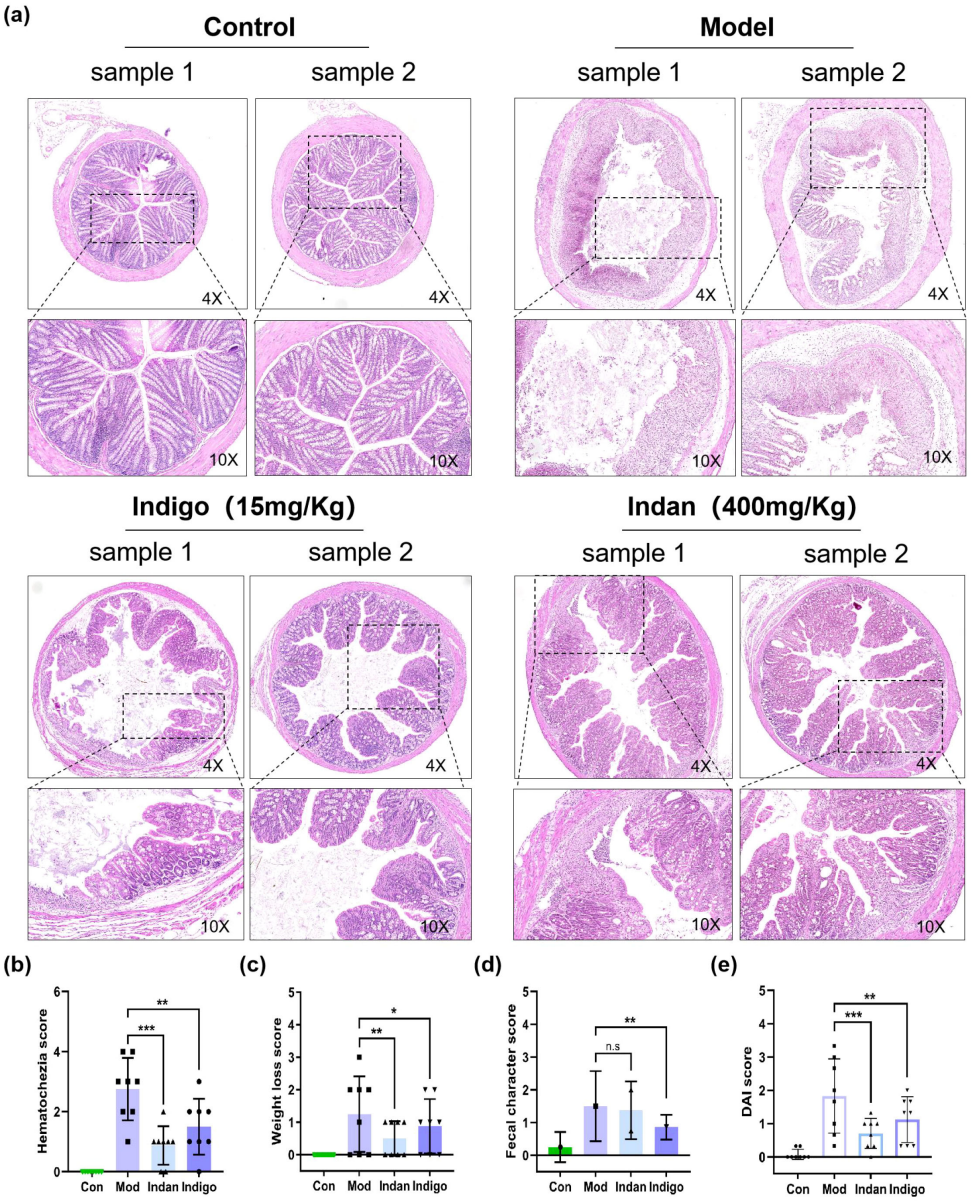
The intervention of Indan/Indigo improved the colonic pathological lesions in mice and reduced the DAI score. **(A)** HE staining results of colonic tissue from each group, images taken at 4× and 10× magnification; **(B)** Fecal bleeding score; **(C)** Percentage score of weight loss; **(D)** Fecal viscosity; **(E)** DAI score. *p < 0.05,**p < 0.01, ***p < 0.001.

Colonic histopathological changes are an important diagnostic indicator of UC, and the pharmacodynamic effects of Indan and Indigo in treating UC were investigated through pathological observation and clinical symptom assessment. In the Control group, no pathological changes were observed in the muscularis mucosae, submucosa, muscular layer, and serosa, as shown in **Figure 2A**, upper left. In the Model group, visible mucosal layer, epithelial cell damage, and tight junctions are destroyed. The intestinal crypt structure is disordered, even completely disappeared. The lamina propria is densely infiltrated by a large number of various inflammatory cells. The mucosal muscular layer thickens or is destroyed due to the spread of inflammation. Submucosa shows edema, an increase in the number of inflammatory cells, and vascular dilation, as shown in **Figure 2A**, upper right. Compared with the Model group, after Indigo treatment, the degree of intestinal crypt distortion in the mucosal layer of the Model group was reduced, and the epithelial cell layer was partially repaired. The infiltration of inflammatory cells in the lamina propria decreased. The mucosal muscular layer and submucosal layer showed a trend towards normalization, as shown in **Figure 2A**, lower left. Similarly, the histology of the colon tissue in the Indan group showed significant improvement compared to the Model group. The mucosal layer showed partial repair. The signs of epithelial cell damage decreased. The density of inflammatory cells in the lamina propria decreased. The appearance of the mucosal muscular layer was more regular, and the edema and inflammation of the submucosa were reduced, as shown in **Figure 2A**, lower right. The colon tissue of the Model group mice showed obvious pathological damage, consistent with UC pathological changes, indicating successful modeling. Indan and Indigo treatments both improved the pathological changes in the colon tissue, reduced the degree of inflammation, and promoted tissue repair.

DAI index is an important index for evaluating clinical symptoms of UC. The DAI index of the Model group was significantly increased, and the scores of weight loss rate, stool viscosity and fecal bleeding were higher than those of the Control group. The DAI index, weight loss percentage, and stool bleeding score of the Indan group were all lower than those of the Model group. The DAI index, weight loss percentage, stool viscosity, and stool bleeding score of the Indigo group showed statistically significant differences. Both Indan and Indigo treatments could effectively improve the symptoms of UC, as shown in **Figures 2B-E**.

### 3.2 Alterations in GM diversity trigger the onset of UC

**Figure 3:**
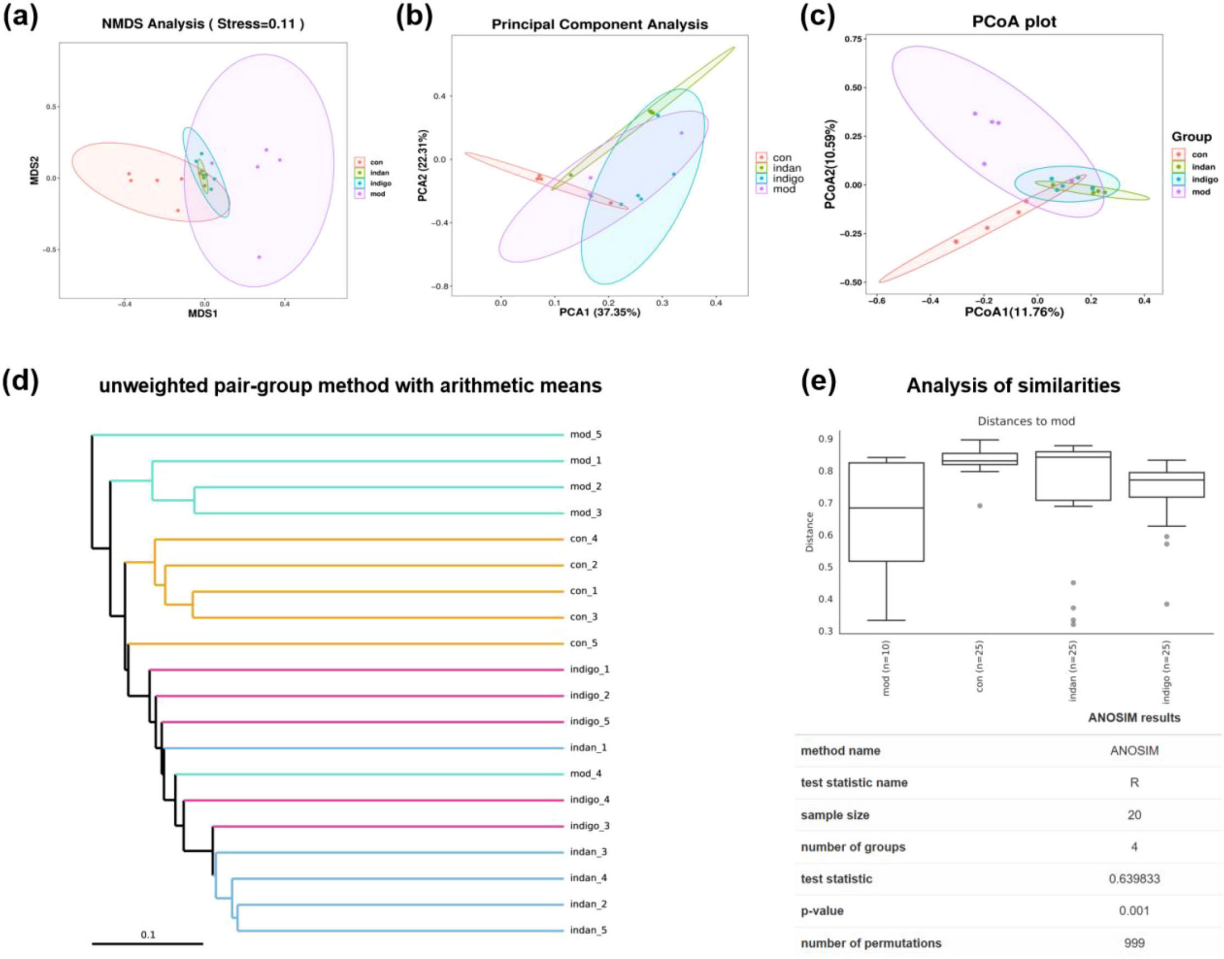
The differential microbiota is related to GM. **(A)** Multidimensional analysis, showing the similarity and difference between groups. The closer the distance between samples, the more similar the composition of the species in the samples; **(B)** Principal component analysis, the distribution of samples in the principal component space, the more similar the composition of the species in the samples, the closer the distance in the PCA diagram; **(C)** PCoA diagram, showing the relationship between samples, the closer the distance between dots, the more similar the structural composition of the species between samples; **(D)** Cluster tree diagram shows the clustering relationship between groups, the color of the branches in the diagram represents the group; The cluster tree shows the similarity between samples, the shorter the branches between samples, the more similar the two samples; **(E)** Anosim similarity analysis, the closer the R value is to 1, the greater the difference between groups, the smaller the difference within groups, and the better the grouping effect; R = 0 indicates that the grouping effect of the samples is equivalent to random allocation, and there is no statistical difference between the grouping of the samples; R = Negative value represents that the difference within the group exceeds the difference between the groups, and the grouping effect of the samples is poor. *p* < 0.05, statistically significant.

Alterations in microbial diversity are a significant factor in the onset of UC. NMDS analysis results show a certain degree of separation among all groups, with the best aggregation of samples within the Indan group, and convergent aggregation areas appearing between the Indan and Indigo groups (**Figure 3A**). PCA analysis showed that the contribution values of PC1 and PC2 were 37.35% and 23.31%, respectively, with significant separation of microorganisms among groups, and good intra-group aggregation in both the Control and Indan groups (**Figure 3B**). The contribution values of PCoA1 and PCoA2 were 11.76% and 10.95%, respectively, similar to the NMDS results (**Figure 3C**). The cluster tree diagram shows that compared to the Model group, the Indan group, Indigo group, and Control group exhibit higher microbial homology and more similar biological characteristics among the groups (**Figure 3D**). Analysis of similarities showed that the test statistic value (R value) = 0.639833, *p* = 0.001. The results indicated that the composition of microbial communities among samples had significant differences between groups and minor differences within groups, with distinct microbial category variations among different groups (**Figure 3E**). The analysis of the differential diversity of colonic microbiota shows that there is a significant difference in the microbial community between the Model group and the other groups. The GM composition of UC lesion mice showed significant differences compared to the normal group. Indan and Indigo altered the composition of GM in mice with UC lesions.

### 3.3 The structural and compositional changes in GM induce UC

**Figure 4:**
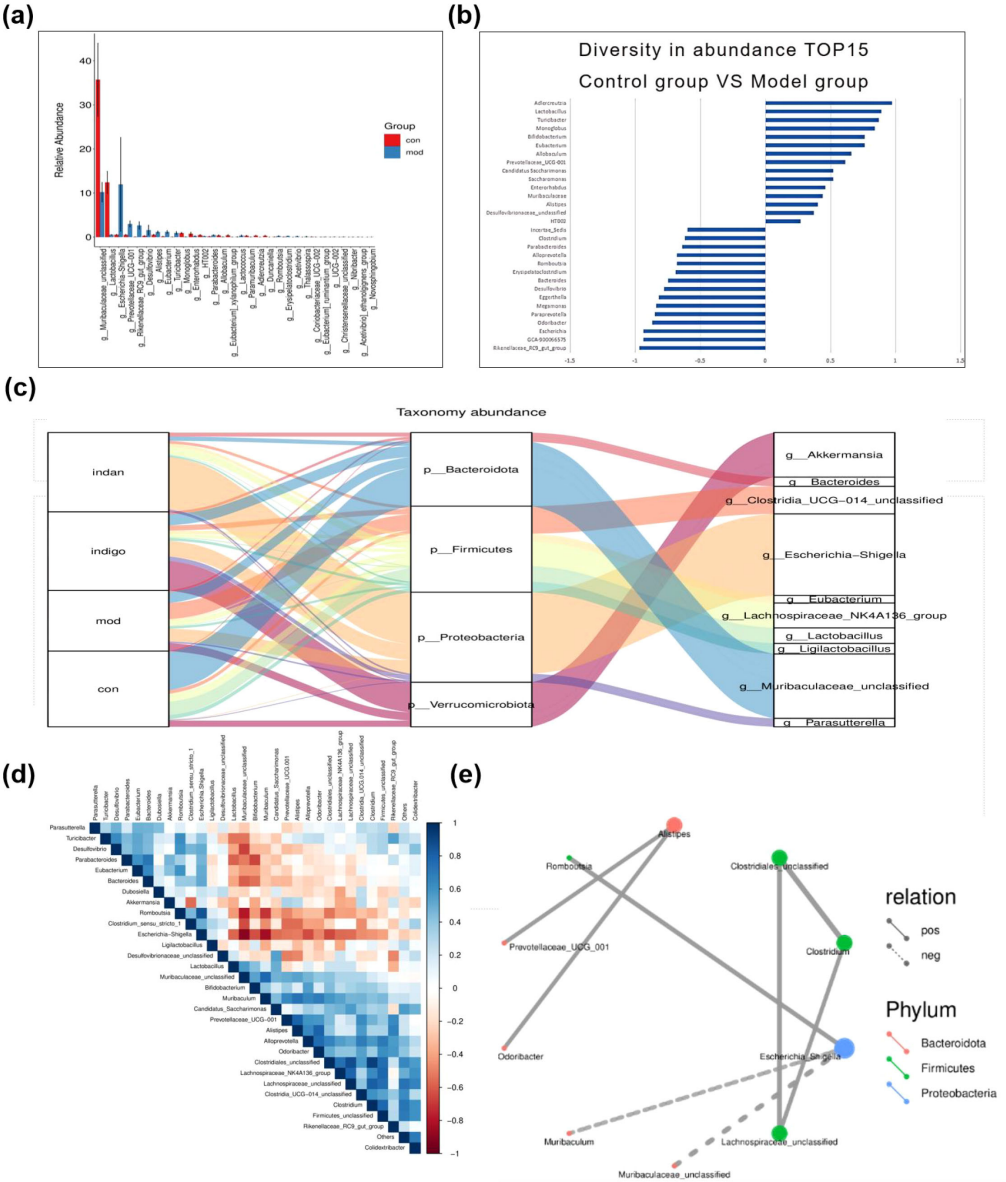
Changes in abundance and structure of microbiota regulate the UC process. **(A)** Barplot difference analysis (taxonomic level), bar chart of the top 10 species with high abundance. The horizontal axis represents the differential species (arranged from left to right by abundance), and the vertical axis is the relative abundance; **(B)** The abundance change rate of bacterial genera, the horizontal axis is the change rate of bacterial genera, and the vertical axis is the bacterial genus category. The larger the absolute value of the change rate, the higher the difference. Change rate > 0 indicates the dominant genera of the Control group, and change rate < 0 indicates the dominant genera of the Model group; **(C)** Sankey diagram shows the composition and proportion relationship of microorganisms at different classification levels (phylum and genus); **(D)** Spearman heat map; **(E)** Spearman network map, filtering for pairs with correlation coefficient |rho| > 0.8. Node color represents the phylum to which the species belongs, line thickness represents the strength of correlation, solid lines indicate positive correlation, and dashed lines indicate negative correlation. Thicker lines represent stronger correlation, while thinner lines represent weaker correlation. Node size indicates the number of other bacterial genera associated with the genus. The more associations, the larger the node; conversely, the smaller the node.

The structural and compositional changes in the GM across different groups promote the development of UC lesions. First, we analyzed two subsets of bacterial genera: highly abundant colon genera and those exhibiting significant intergroup abundance variation in mice. Bar plot analysis identified the top 10 abundant genera with significant differences between the control and model groups as the core differential genera: *Muribaculaceae_unclassified, Lactobacillus, Escherichia-Shigella, Prevotellaceae_UCG-001, Rikenellaceae_RC9_gut_group, Desulfovibrio, Alistipes, Eubacterium, Turicibacter* and *Monolobus* (**Figure 4A**). Afterwards, we analyzed the structural changes among the groups. *Muribaculaceae_unclassified*, the dominant colonic genus in mice, was significantly more abundant in the control group, implying its depletion may drive UC development. *Lactobacillus, Alistipes, Eubacterium* and *Turicibacter* were enriched in the control group (suggesting intestinal protective effects), while *Escherichia, Prevotellaceae_UCG-001, Rikenellaceae_RC9_gut_group* and *Desulfovibrio* were elevated in the model group (indicating potential UC-inducing roles). Genera with marked intergroup abundance variation also contributed to UC lesions. Compared with the model group, control group genera with increased abundance (ranked by change rate) were *Adlercreutzia, Lactobacillus, Turicibacter, Monoglobus, Bifidobacterium, Eubacterium, Allobaculum* and *Prevotellaceae_UCG-001*; those with decreased abundance were *Rikenellaceae_RC9_gut_group, Escherichia, GCA-900066575, Odoribacter, Paraprevotella, Megamonas, Eggerthella* and *Desulfovibrio* (**Figure 4B**).

Microbial abundance shifts induced structural remodeling and intergroup heterogeneity in dominant microbiota. Control group: Bacteroidota (dominant, with *Muribaculaceae_unclassified* as core genus) and Firmicutes were dominant phyla; *Lactobacillus* and *Lachnospiraceae_NK4A136_group* dominated Firmicutes. Model group: No dominant phyla; Firmicutes and Proteobacteria were slightly more abundant, with *Clostridia_UCG-014* (Firmicutes) and *Escherichia* (Proteobacteria) as core genera. Indigo group: No dominant phyla; Verrucomicrobiota and Proteobacteria were slightly more abundant, with *Akkermansia* (Verrucomicrobiota) and *Escherichia* (Proteobacteria) as core genera. Bacteroidota (*Muribaculaceae_unclassified*, *Bacteroides*) and Firmicutes (enhanced diversity, core genera: *Lachnospiraceae_NK4A136_group*, *Clostridia_UCG-014, Ligilactobacillus*) were also present. Indan group: Proteobacteria and Firmicutes were dominant phyla, with *Escherichia* (Proteobacteria) and *Lachnospiraceae_NK4A136_group* (Firmicutes) as core genera (**Figure 4C**).

Phylogenetic tree analysis showed Firmicutes and Bacteroidota had complex evolutionary branches with high genus diversity, while Proteobacteria and Verrucomicrobiota had simple branches with low diversity (**Supplementary Figure 1A**). Representative colonic microbiota exhibited group-specific abundance patterns: extremely low *Escherichia* and dominant *Muribaculaceae_unclassified* (control); dominant *Akkermansia* (Indigo); dominant *Clostridia_UCG-014* (model); dominant *Lachnospiraceae_NK4A136_group* (Indan) (**Supplementary Figure 1B**).Control mice GM was dominated by Bacteroidota and Firmicutes, whose significant depletion in UC mice indicated a key role in UC induction. *Escherichia* was significantly enriched in all groups except the control, confirming it as a pivotal UC pathogenic genus. Compared with the model group, Indan and Indigo groups increased colonic microbial diversity and probiotic abundance, while reducing harmful bacteria: Indigo upregulated *Akkermansia*; Indan elevated Firmicutes proportion and suppressed *Clostridia_UCG-014* within it. Both treatments remodeled colonic GM structure in UC mice.

UC-related genera exhibited competitive and symbiotic interactions (**Figure 4D**). Spearman correlation analysis revealed significant positive abundance correlations between *Alistipes* and *Prevotellacae_UCG_001, Romboutsia* and *Escherichia, Lachnospiraceae_unclassified* and *Clostridiales*; significant negative correlations between *Muribaculum/Muribaculaceae_unclassified* and *Escherichia* (**Figure 4E**). *Muribaculaceae_unclassified* and *Escherichia* are key for intestinal health, and their dynamic balance is critical for UC onset.

### 3.4 Indan regulates key gut bacterial genera to improve UC lesions

**Figure 5:**
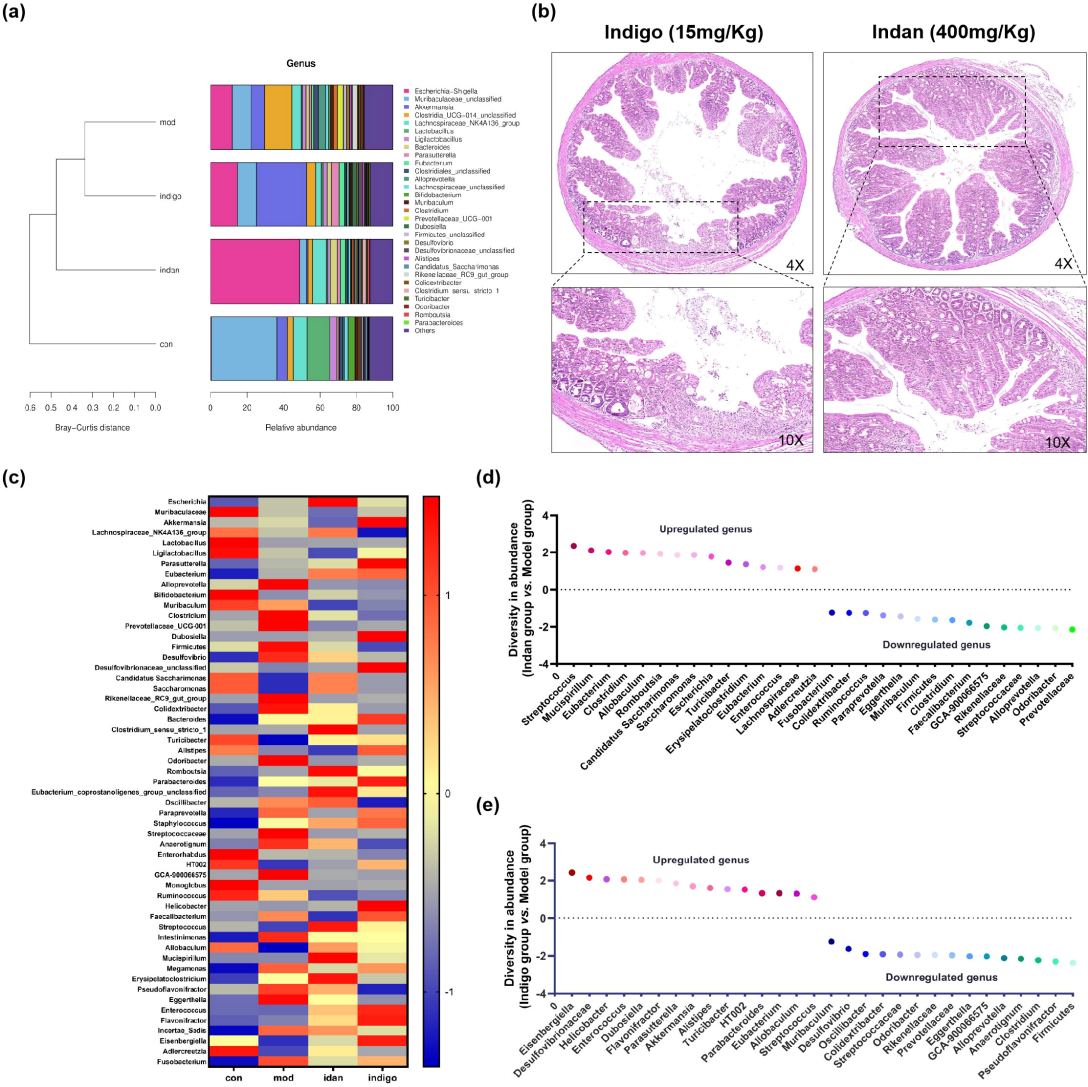
Indan/Indigo regulation of GM abundance in the treatment of UC. **(A)** Left: Bray-curtis distance clustering tree structure, the closer the samples are clustered, the shorter the branches, indicating more similar species composition. Right: Relative abundance distribution of species at the genus level, the larger the proportion indicates higher abundance; **(B)** HE staining image of mouse colon, images taken at 4× and 10× magnification; **(C)** Heatmap showing the differences in relative abundance of genera between groups; **(D)** Change rate of genus abundance chart (Indan group compared to Model group); **(E)** Change rate of genus abundance chart (Indigo group compared to Model group). The vertical axis represents the change rate of genera between the two groups, the horizontal axis represents the genus category, the larger the absolute value of the change rate indicates higher difference, the change rate > 0 indicates up-regulated abundance, and the change rate < 0 indicates down-regulated abundance.

The diversity and structure of GM are key factors in UC lesions, and we analyzed the regulatory effects of Indan and Indigo on key gut bacterial genera associated with UC. Bray-curtis analysis showed that there were differences in genera between groups, with the microbial structure of the Indan group most similar to the Control group (**Figure 5A**). After treatment with Indan and Indigo, the colonic wall tissue structure of mice was restored, and inflammatory lesions were weakened (**Figure 5B**). This indicates that Indan/Indigo improves UC lesions by regulating GM abundance.

Indan and Indigo altered the abundance of SCFAs, inflammatory immunity, and angiogenesis related bacterial genera. The results show that Indan can increase the abundance of bacterial genera, such as *Lachnospiraceae_NK4A136_group*, *Eubacterium*, *Candidatus_Saccharimonas*, *Clostridium_sensu_stricto_1*, *Bifidobacterium*, *Turicibacter*, *Adlercreutzia*. *Eubacterium, Turicibacter* and *Allobaculum.* Indan can reduce the abundance of *Prevotella*, *Eggerthella*, *Clostridium*. *Rikenellaceae_RC9_gut_group Pseudoflavonifractor* and *Desulfovibrio.* Indigo can increase the abundance of bacterial genera such as *Eubacterium*, *Turicibacter*, *Adlercreutzia*, *Candidatus_Saccharimonas*, *Ligilactobacillus*, *Alistipes*, *Akkermansia*, *Eubacterium Turicibacter* and *Allobaculum*.Indigo can reduce the abundance of *Prevotella*, *Eggerthella*, *Clostridium, Rikenellaceae_RC9_gut_group Pseudoflavonifractor* and *Desulfovibrio* (**Figure 5C**).

Indan with Indigo changed the abundance of many other bacterial genera. Compared with the Model group, the genera with a higher abundance increase rate in the Indan group were *Streptococcus*, *Mucispirillum*, *Romboutsia*, *Saccharomonas*, *Escherichia*, *Erysipelatoclostridium*, *Enterococcus*; the genera with a higher abundance decrease rate were *Odoribacter*, *Peptostreptococcaceae_unclassified*, *GCA-900066575*, *Faecalibacterium*, *Muribaculum*, *Ruminococcus*, *Colidextribacter*, *Fusobacterium*, as shown in **Figure 5D**. The genera with a high abundance adjustment rate are *Eisenbergiella*, *Desulfovibrionaceae_unclassified*, *Helicobacter, Enterococcus*, *Dubosiella*, *Flavonifractor*, *Parasutterella*, *HT002*, *Parabacteroides*, *Streptococcus*; the genera with a high abundance adjustment rate are *Anaerotignum*, *GCA-900066575*, *Odoribacter*, *Peptostreptococcaceae_unclassified*, *Colidextribacter*, *Oscillibacter*, *Muribaculum*, as shown in **Figure 5E**. There are competitive and symbiotic relationships among genera, and the genera with higher abundance change rates mentioned above may be potential factors for the reversal of UC lesions by Indan.

### 3.5 The high abundance of beneficial bacteria increased the content of SCFAs

**Figure 6:**
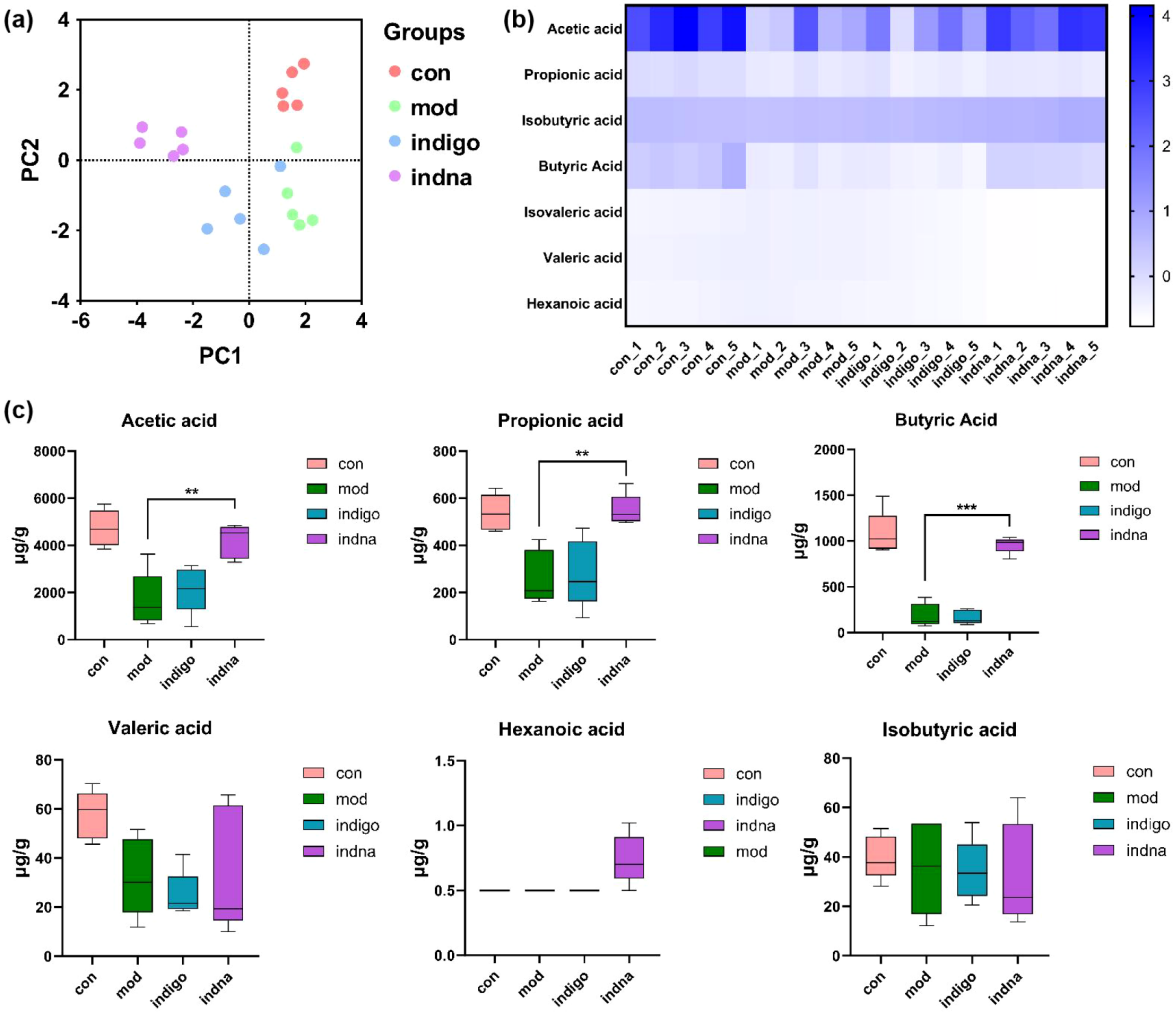
SCFAs are the key GM metabolites in the pathogenesis and treatment of UC. **(A)** PCA analysis of SCFAs, different colored dots represent different groups, the closer the sample points are, the higher the similarity between samples; **(B)** Heatmap of SCFAs clustering, showing the content of SCFAs in each group of mice, the depth of color represents the expression level of SCFA in the sample; **(C)** Boxplot of quantitative analysis of SCFAs, showing the distribution of SCFA content in different groups of mice. The horizontal axis represents the groups, while the vertical axis indicates the content of SCFAs (Acetic acid, propionic acid, butyric acid, valeric acid, hexanoic acid, isobutyric acid). **p < 0.01, ***p < 0.001, Indan group *vs.* Model group.

SCFAs are important metabolic products of GM, playing a key role in intestinal function, body metabolic regulation, and maintaining immune homeostasis. The accumulation of SCFAs can promote the proliferation and differentiation of intestinal epithelial cells, enhance intestinal barrier function, inhibit inflammation, regulate immune cell activity, and change the intestinal pH value. PCA results show that PC1 and PC2 dimensions are significantly separated, with each group having a different cluster area, indicating a significant difference in the SCFAs metabolic profile between UC lesioned mice and normal mice. The intervention of Indan and Indigo changed the SCFAs metabolic profile. The clustering of the Indan group is better than that of the Indigo group, indicating that the sample differences within the Indan group are smaller (**Figure 6A**).

The heatmap and boxplot show that compared to the Control group, the contents of acetic acid, propionic acid, butyric acid, and valeric acid in the Model group are significantly reduced. Compared with the Model group, the acetate, propionate, and butyrate contents in the Indan group were significantly increased, and the acetate, propionate, and butyrate contents in the Indigo group showed an increasing trend (median increased, but with large variability within the group), but there was no statistical difference. Hexanoic acid was detected in the Indan group (not detected or present at very low levels in other groups). Compared to the Model group, there was no statistical difference in the contents of iso-butyric acid and valeric acid between the Indan group and the Indigo group, and the content of valeric acid in the Indigo group samples was significantly increased (**Figures 6B, C**). The results show that a decrease in SCFAs content induces the onset of UC, both Indan and Indigo treatments can increase SCFAs content, with Indan being superior to Indigo in regulating the stability of SCFAs. SCFAs content correlates with DAI scores and pathological observations. Increased SCFAs levels can reverse UC, trigger SCFAs key GM metabolites in both the pathogenesis and treatment of UC.

### 3.6 Indan increases the abundance of Clostridium-sensu_stricito_1 to produce hexanoic acid for preventing UC recurrence

The production of hexanoic acid by Indan is an important discovery. In UC lesions, acetic acid, propionic acid, and butyric acid have a clear effect on the intestines, and valeric acid also plays an important role in UC. The level of hexanoic acid in patients with recurrent UC is lower (Russo et al., 2022), we have also observed in clinical treatment that Indan can effectively prevent the recurrence of UC. The generation of hexanoic acid may be related to *Clostridium-sensu_stricito_1*. The study shows that *Clostridium-sensu_stricito_1* is a unique microorganism of the Indan group (with extremely low abundance in other groups), as show in **Figure 5C**, and there is research indicating a positive correlation between *Clostridium-sensc_tricto_1* and hexanoic acid (Zhao et al., 2024). Indan increases the abundance of *Clostridium-sensu_stricito_1* to produce hexanoic acid, which may be the mechanism by which Indan prevents UC recurrence.

### 3.7 Indan increases SCFAs content and change bacterial phenotypes

Bacterial phenotypic changes are related to intestinal health, such as the increase in abundance of aerobic and anaerobic affecting the intestinal health status, and the increase in Gram-positive bacteria can cause inflammatory lesions. Besides regulating inflammation and immune responses, changes in the levels of SCFAs also affect environmental factors such as oxygen content and pH in the intestines, thereby altering the distribution of bacterial phenotypes. The results show that compared with the Control group, the abundance of Aerobic significantly increased in the Model group; compared with the Model group, the abundance of Aerobic significantly decreased in the Indan group. In the Model group and indigo group, Aerobic is mainly composed of Verrucomicrobia microorganisms, while the Indan group is mainly composed of other types of microorganisms (**Supplementary Figure 2A**). Compared to the Model group, the abundance of Anaerobic organisms increased in the Indan group and decreased in the Indigo group. In terms of phyla, the Anaerobic microorganisms showed differentiated distributions among the groups. The Bacteroidetes microbial abundance was highest in the Control group, while the Firmicutes microbial abundance was highest in the Model group and the Indigo group, and the abundance of other types of microorganisms was highest in the Indan group (**Supplementary Figure 2B**). Compared with the Control group, the abundance of facultatively anaerobic microorganisms increased in the other groups, with the highest abundance of Proteobacteria (**Supplementary Figure 2C**). For Gram-negative and Gram-positive microorganisms, the change trend of the Indigo group is similar to that of the Control group (**Supplementary Figures 2D, E**). In the Model group, Indigo group, and Indan group, the abundance of Proteobacteria microorganisms is the highest (**Supplementary Figure 2F**). The results show that Indan can significantly inhibit aerobic abundance. The differential regulation of aerobic abundance by Indan and Indigo is related to the content of SCFAs, which can reduce intestinal oxygen content and inhibit aerobic abundance. The content of SCFAs is negatively correlated with aerobic abundance. In Faculty anaerobic, both Indan and Indigo increased the abundance of Proteobacteria compared to the model group, while suppressing the abundance of other bacterial genera.

Peptidoglycan is a specific signal for bacterial host immune recognition and plays a role in enhancing immunity. The composition of peptidoglycan can reach 50~80% in the cell wall of Gram-positive bacteria, while it only accounts for 5~20% in the cell wall of Gram-negative bacteria. Peptidoglycan can stimulate mononuclear macrophages and endothelial cells to release immune regulatory substances, such as TNF-α, interleukin, and interferon, causing an inflammatory response. Data shows that compared to the Control group, the abundance of Gram-negative bacteria in the Model group decreased, while the abundance of Gram-positive bacteria increased. The increase in the proportion of Gram-positive bacteria may be one of the causes of UC. The abundance of Gram-positive bacteria in the Indigo group was significantly reduced, approaching that of the Control group. Inhibiting the abundance of Gram-positive bacteria is one of the mechanisms by which Indigo improves UC, as shown in **Supplementary Figure 2D, E**.

### 3.8 GM changes lead to alterations in biological function

Changes in GM abundance that lead to alterations in biological functions are the direct cause of UC lesions. We analyzed the differences in biological functions between groups. Modeling induces changes in biological functions, which are reflected in enzyme activity. The microbial community in the control group enhances the activity of Dipeptidase, Hydroxymethylpyrimidine pyrophosphatase, HAD family phosphatases, and Aminopeptidase C. The microbial community in the Model group enhances the activity of Enoyl-CoA hydratase/carnitine racemase and 3-hydroxyacyl-CoA dehydrogenase (**Supplementary Figure 3A**). The Indan group and the Indigo group have different biological functions. The main function of the GM in the Indan group is to activate the Bifunctional DNA-binding transcriptional regulator of the YhaV-PrlF toxin-antitoxin module, while Indigo activates the Membrane protein insertase Oxa1/YidC/SpoIIIJ (**Supplementary Figure 3B**).

The Indan and Indigo treatment groups also showed differences in biological function. Compared with the Model group, Indan inhibits the activity of Nucleoside-diphosphate-sugar epimerase, Methylmalonyl-CoA mutase, Formylglycine-generating enzyme, and sulfatase; Indan activates the activity of Fe^3+^-transporting ATPase, ATPase, Ser/Thr protein kinase. Enhances the antagonistic effect of MazF, and promotes the expression of DNA-binding transcriptional regulators (**Supplementary Figure 4A**). Compared with the Model group, Indigo inhibits the maturation enzyme AslB, SAM, Fe^3+^-hydroxamate activity, and activates the arabinose efflux permease, MFS family, and hydrogenase maturation factor activity (**Supplementary Figure 4B**).

### 3.9 Specific bacterial genera are expected to become biomarkers for UC treatment

**Figure 7:**
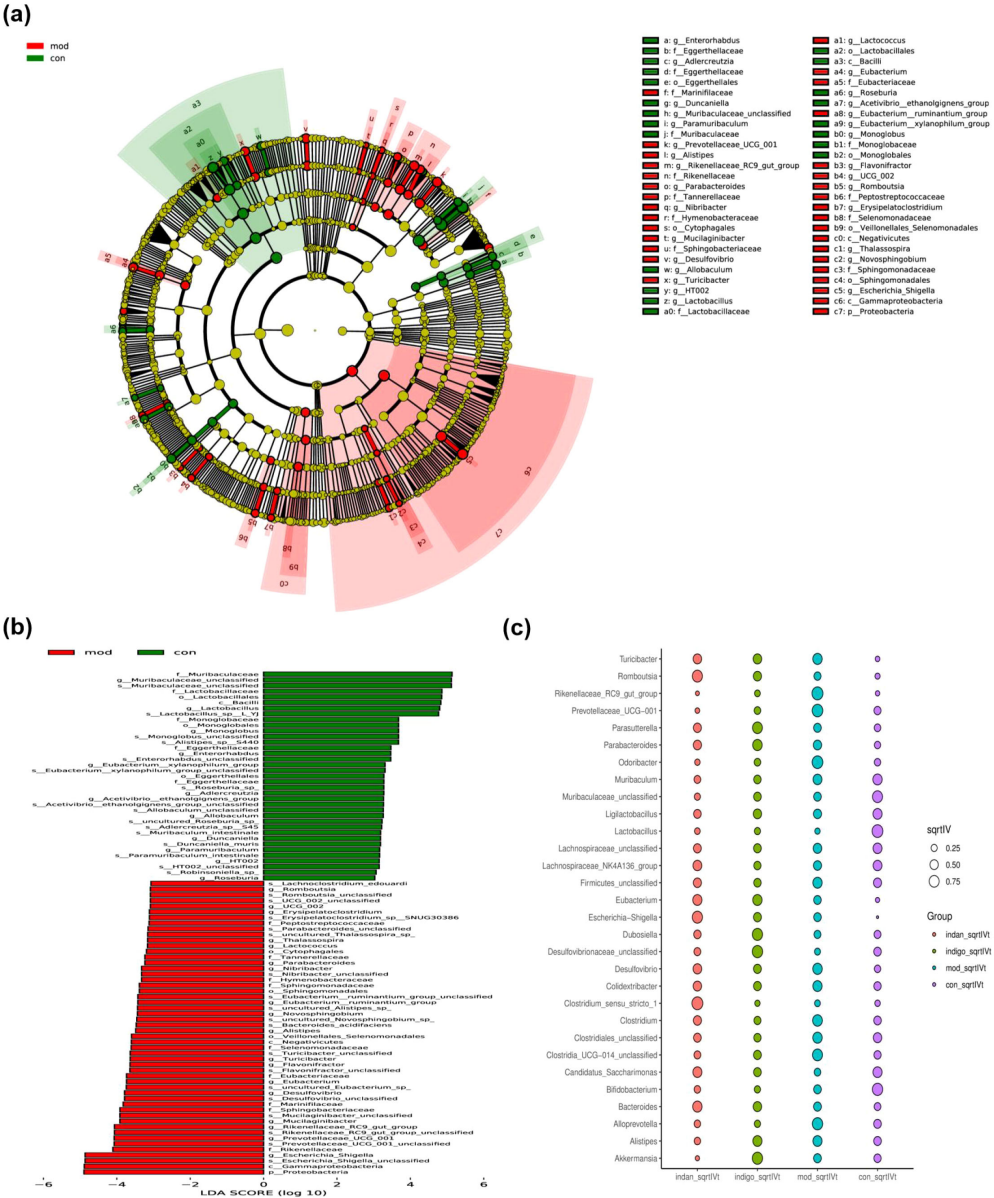
The GM with significant changes is a biomarker for UC diagnosis. **(A)** Microbial community structure: The circles from the inside out represent different taxonomic levels of microorganisms (from phylum to species), with node size indicating species abundance. Yellow nodes represent no significant difference in abundance between the Model group and the Control group for that species; red nodes represent higher abundance in the Model group than in the Control group; green nodes represent higher abundance in the Control group than in the Model group. **(B)** LDA score histogram: Shows the microorganisms and their scores that contribute significantly to the difference between the two groups. Red bars represent important microbial communities in the Model group; green bars represent important microbial communities in the Control group. **(C)** Indicator analysis: The sqrtIVt value is the square root of the indicator value, and the higher the value, the more likely the species is to serve as a biomarker for the treatment group.

GM alteration induced UC lesions, and the significantly changed microbial community is expected to serve as a biomarker for UC lesions. The structure of the microbial community (circular diagram) shows that Firmicutes undergo differential evolutionary branches between the Control group and the Model group. At the class level, compared with the Model group, the abundance of Bacilli was significantly increased in the Control group; at the order level, *Lactobacillus* and *HT002* were the dominant microbial groups in the Control group, while *Lactococcus* was the dominant microbial group in the Model group. Additionally, compared to the Control group, the abundance of Proteobacteria in the Model group was significantly increased, with *Escherichia*, *Novosphingobium*, and *Thalassospira* being the dominant microorganisms (**Figure 7A**). LDA analysis shows that Firmicutes and Bacteroidetes are the dominant phyla in the control group. Within Firmicutes, the dominant microbial groups include Bacilli (class), Lactobacillales (order), Lactobacillaceae (family), *Lactobacillus* (genus), and *Lactobacillus* sp. *L_YJ* (species). Within Bacteroidetes, the dominant groups include Muribaculaceae (family), *Muribaculaceae_unclassified* (genus), and *Muribaculaceae_unclassified* (species), are dominant microbial groups. In the Model group, Proteobacteria is the dominant phylum, with Gammaproteobacteria (family), *Escherichia_Shigella* (genus), and *Escherichia_Shigella_unclassified* (species) as the dominant microbial groups (**Figure 7B**). *Rikenellaceae_RC9_gut_group*, *Odoribacter*, *Prevotellaceae_UCG-001*, *Alloprevotella*, *Clostridia_UCG-014_unclassified* are the main indicators of the Model group. *Lactobacillus*, *Bifidobacterium*, *Muribaculaceae_unclassified*, *Muribaculum*, *Candidatus_Saccharimonas* are the main indicators of the Control group (**Figure 7C**). The results indicate that the abundance of Proteobacteria and *Escherichia* has increased, while the abundance of Firmicutes, Bacteroidetes, *Lactobacillus*, and Muribaculaceae has decreased, suggesting the possibility of UC lesions. The abundance of the main indicator bacterial genus decreased in the Control group, indicating an increased risk of UC; the abundance of the main indicator bacterial genus increased in the Model group, indicating a more severe UC lesion.

## 4 Discussion

**Figure 8:**
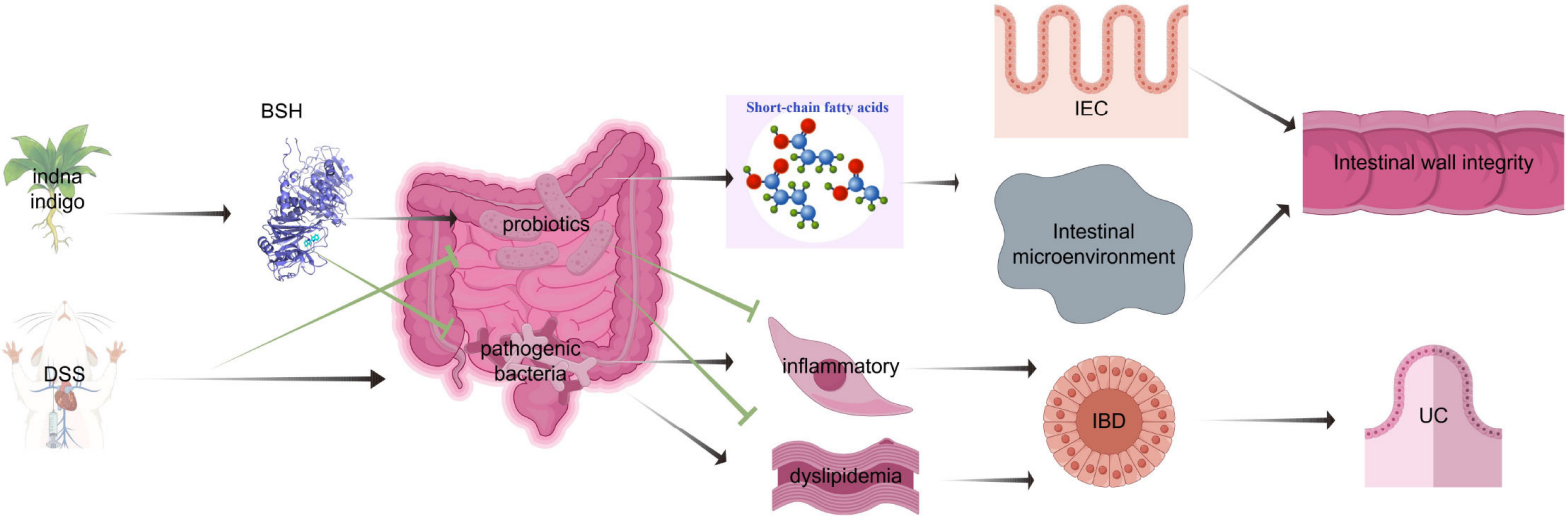
Indan/Indigo regulation GM to reverse UC. Indan/Indigo inhibits the growth of harmful bacteria, promotes the proliferation of probiotics, increases the content of SCFAs, provides energy for the repair of intestinal epithelial cells, alters the intestinal microenvironment, inhibits inflammatory reactions and lipid metabolism disorders, regulates angiogenesis, and treats UC.

Studies have confirmed the relationship between UC lesions and GM, with a specific focus on the interplay between these two factors (Sankarasubramanian et al., 2020). Our research shows that changes in GM abundance and structure, under the influence of competition and symbiotic interactions among microorganisms, lead to UC lesions. Indan/Indigo regulate GM abundance and structure, repairing the integrity of the intestinal wall to treat UC (**Figure 8**). *Escherichia* is an important bacterial genus that causes UC. After Indan intervention, even with a high abundance of *Escherichia* in the genus, there was a significant improvement in both the clinical symptoms and pathological conditions of UC. The research results suggest that as the disease progresses, although *Escherichia* is a trigger for UC, it is not the main trigger for maintaining symptoms and continuously damaging the intestinal wall. The abundance and structure of GM after UC lesions are altered under Indan and Indigo treatment, affecting GM metabolites. Indan and Indigo exhibit differences in the regulation level and mechanism of SCFAs. Compared to Indigo, Indan shows a higher stability in regulating GM. *Oscillibacter* is one of the main bacterial genera that produce butyric acid, and Indigo has a significant inhibitory effect on *Oscillibacter* (Tian et al., 2021), which may be the reason why Indan is more effective than Indigo in increasing SCFAs content.

The bile salt hydrolase (BSH) activity in the GM of IBD patients is reduced (Duboc et al., 2013). BAs are important regulatory points in the dynamic microbial network (Gadaleta et al., 2022), which can regulate the composition of the BAs pool. In our research related to this experiment, we found that indigo can target BSH and regulate its activity. BSH can hydrolyze amide bonds, converting conjugated bile acids into free amino acids and free bile acids, which are further metabolized into secondary BAs under the action of GM, thereby altering the composition of the bile acid pool. High-level secondary Bas (deoxycholic acid, DCA) has antibacterial activity. Primary Bas (cholic acid, CA) can increase the abundance of opportunistic pathogens (*Prevotella*, *Desulfovibrio*) while reducing the abundance of beneficial bacteria (including *Ruminococcus*, *Lactobacillus*, *Roseburia*). Under low levels of BSH, the level of primary BAs is higher, while the level of secondary BAs is lower (Lloyd-Price et al., 2019). Studies have shown that moderate supplementation of CA helps to increase the abundance of *Clostridia* and *Erysipelotrichi* (Islam et al., 2011), while supplementation of secondary BAs can reduce the abundance of *Clostridia* (Xu et al., 2021). Similar results were also presented in this study, where Indigo activation of BSH suppressed the abundance of *Clostridia*.

Studies have shown that DCA, lithocholic acid (LCA), promote inflammatory response and GM dysregulation. However, in another study, rectal instillation of DCA/LCA in mice produces anti-inflammatory effects (Sinha et al., 2020). Under different experimental conditions and administration methods, secondary BAs have both anti-inflammatory and pro-inflammatory effects, similar to the bidirectional nature of Indan treatment for UC observed in clinical practice (Xu et al., 2024). Indan can treat UC and promote the healing of ulcers. However, there have also been cases of individuals without UC lesions who developed UC after taking Indan. This may be due to the different effects of Indan in different intestinal environments (with or without UC lesions) after it induces the generation of secondary BAs. In future research, confirmatory studies can be conducted in this direction. The biological transformation relationship between GM and BAs has a crucial biological significance (Gadaleta et al., 2022).

## 5 Conclusion

Alterations in the abundance and composition of GM induce UC lesions. The decrease in beneficial bacteria and the increase in harmful bacteria lead to reduced SCFAs levels and an elevated inflammatory response. Indan and Indigo increased the abundance of beneficial bacteria, decreased the abundance of harmful bacteria, elevated the content of SCFAs, and modified bacterial phenotypes, thereby improving UC lesions. Indan can significantly increase hexanoic acid content, and its mechanism may be related to the increase in the abundance of *Clostridium-sensu_stricito_1*. This may be a specific mechanism of Indan in inhibiting the recurrence of UC.

## Data Availability

The raw data supporting the conclusions of this article will be made available by the authors, without undue reservation.
