# Supplementary material for: Indigo Naturalis regulates the gut microbiota to increase SCFAs content and improve ulcerative colitis lesions
Source: Front Cell Infect Microbiol. 2026 Mar 31;16:1772977. doi: 10.3389/fcimb.2026.1772977 (PMC13076306; doi:10.3389/fcimb.2026.1772977)
Supplement: Supplementary file 1 [file Table1.docx]

Supplementary Figures


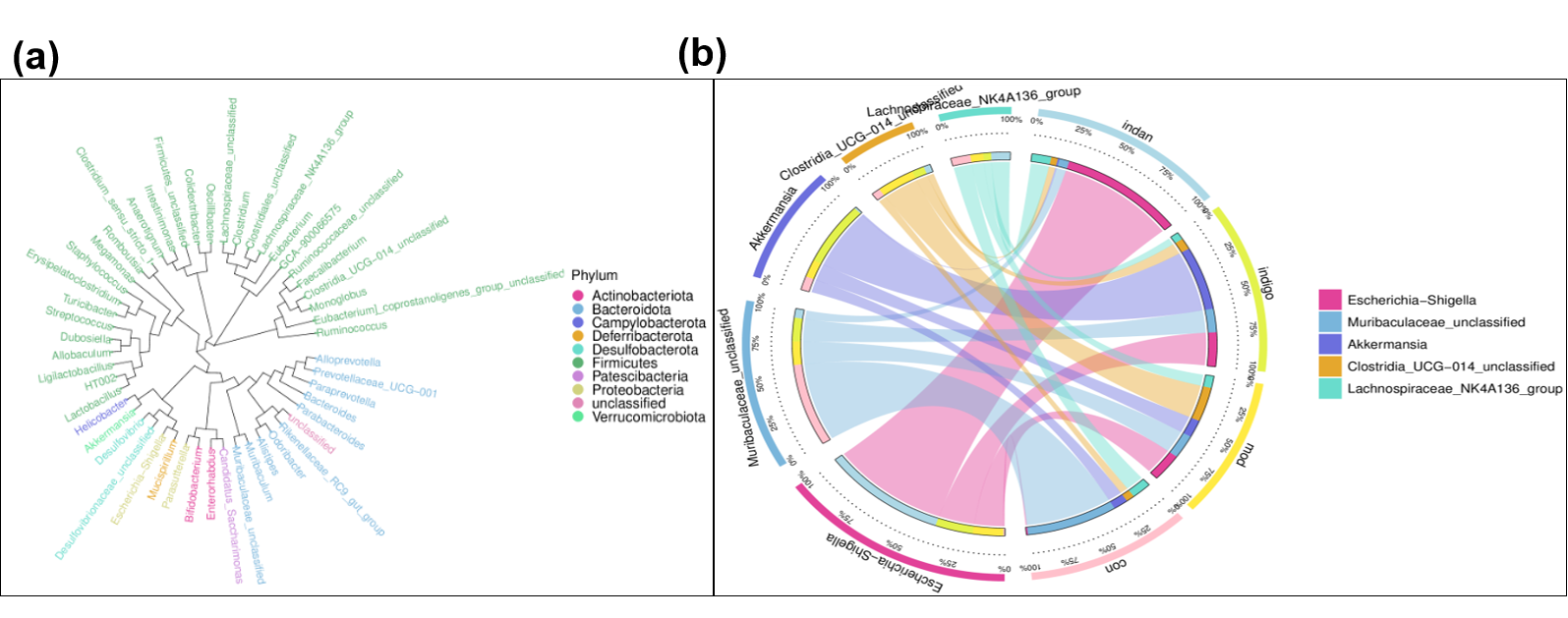


**Supplementary Fig.1 Evolution of gut microbiota composition** (a) Species phylogenetic tree, drawing the top 50 abundant genera, different branches in the figure represent different genera classification, genera are different but colors are the same, indicating that they belong to the same phylum, the closer the distance between two species, the closer their evolutionary relationship; (b) The circular diagram shows the proportion relationship of the top 5 abundant bacterial genera in each group, the wider the width, the higher the abundance.

**
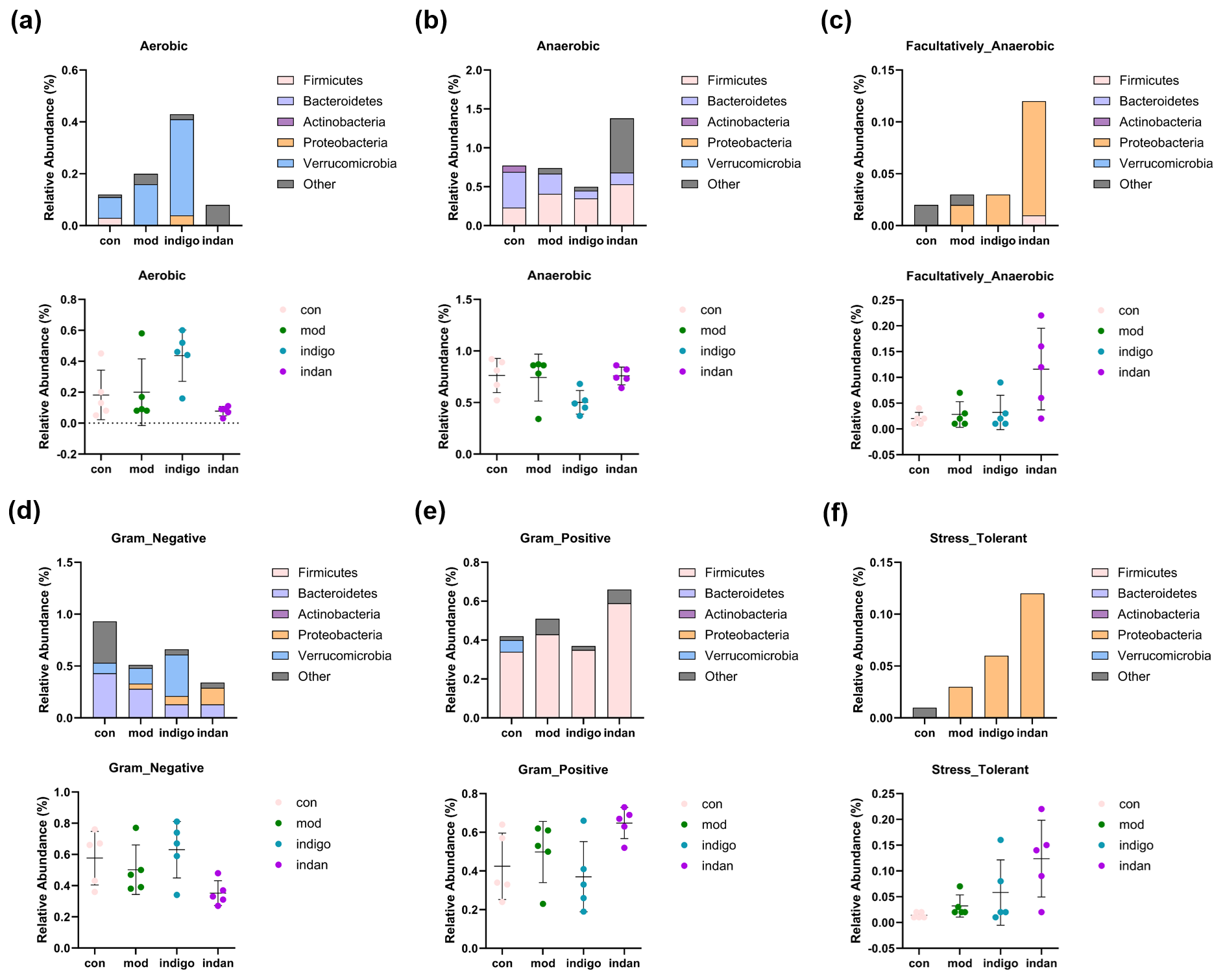
**

**Supplementary Fig.2 SCFAs alter the gut microbiome environment, causing changes in microbial abundance and structure.** (a) Aerobic; (b) Anaerobic; (c) Facultatively Anaerobic; (d) Gram Negative; (e) Gram Positive; (f) Stress_Tolerant. The histogram shows the relative abundance of different phenotypic species in each group, with species distinguished by color; the scatter plot shows the relative abundance of each sample in the differential phenotype in each group, with dots representing samples and color representing groups.

**
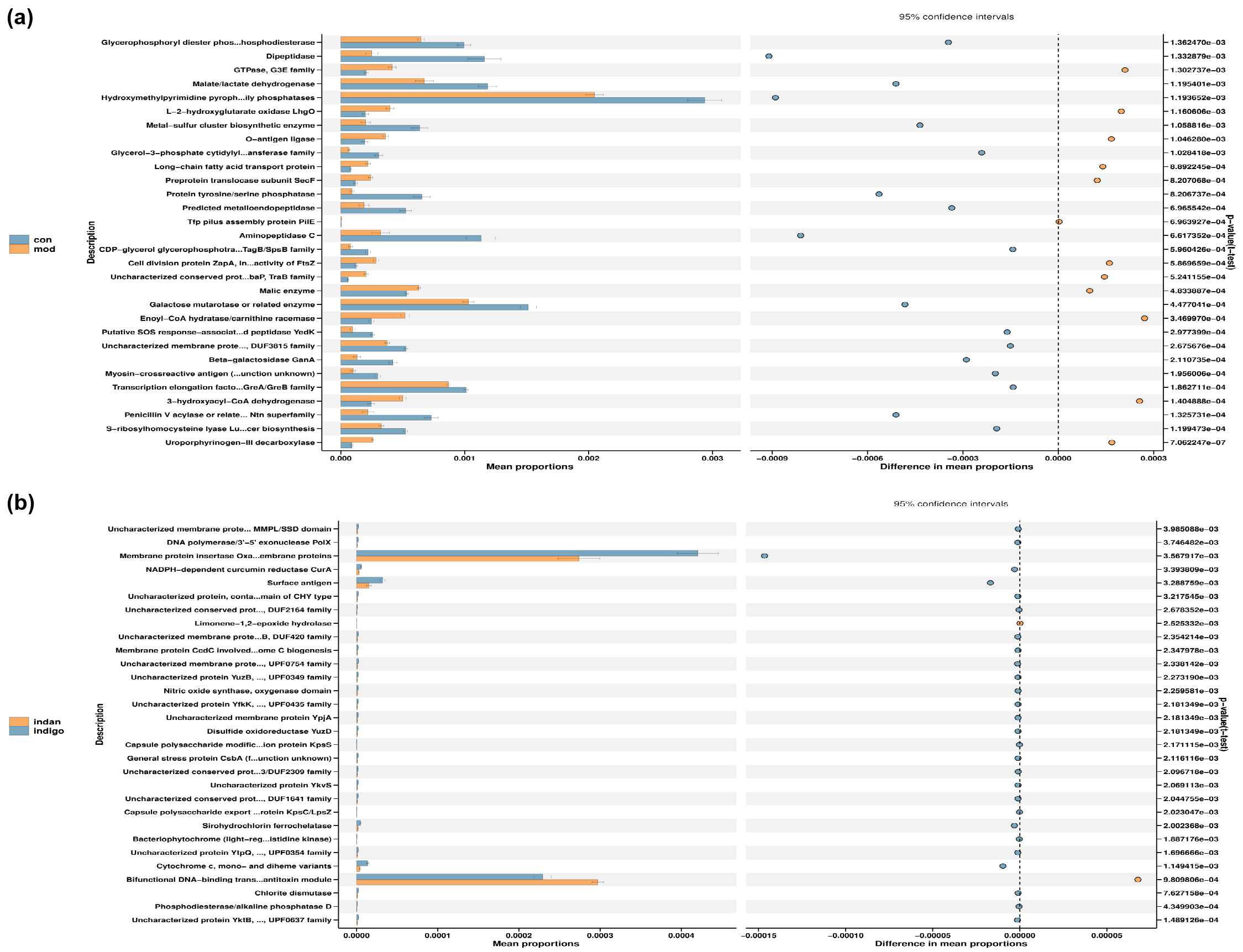
**

**Supplementary Fig.3 Abundance and structural changes of GM lead to changes in biological function.** (a) Biological functional differences between the Control group and the Model group; (b) Biological functional differences between the Indan group and the Indigo group. PICRUSt functional prediction, showing the top 30 functions with *p*<0.05 in the t-test results, 95% confidence interval, predicting the relationship between microbial community and function.

**
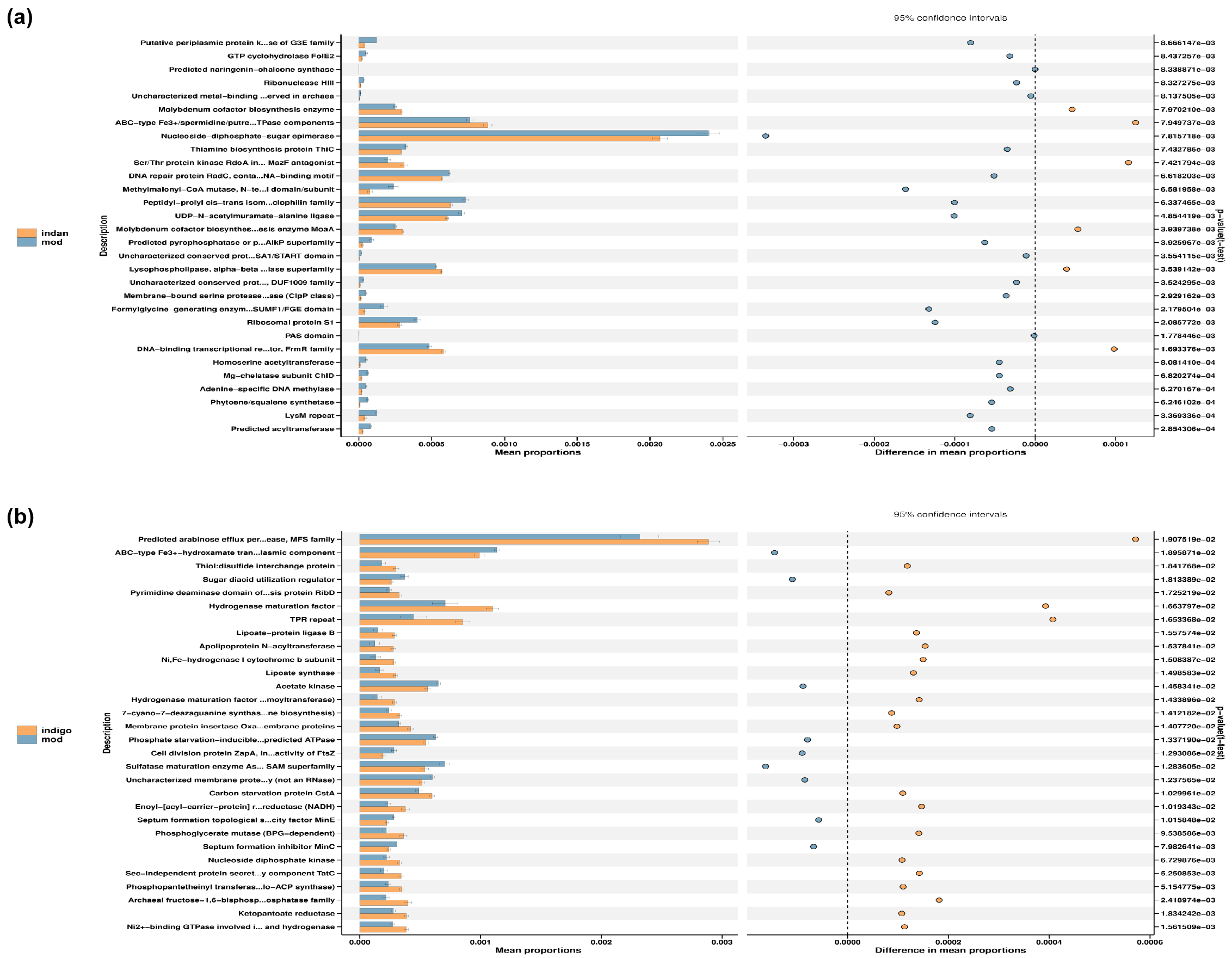
**

**Supplementary Fig.4 GM changes in Indan/Indigo-regulated UC lesion mice and their biological functions.** (a) Biological functional differences between the Control group and the Model group; (b) Biological functional differences between the Indan group and the Indigo group. PICRUSt functional prediction, showing the top 30 functions with *p* < 0.05 in the t-test results, 95% confidence interval, predicting the relationship between microbial community and function.
